# Divergent endothelial mechanisms drive arteriovenous malformations in Alk1 and SMAD4 loss-of-function

**DOI:** 10.1101/2025.01.03.631070

**Published:** 2025-01-03

**Authors:** Olya Oppenheim, Wolfgang Giese, Hyojin Park, Elisabeth Baumann, Andranik Ivanov, Dieter Beule, Anne Eichmann, Holger Gerhardt

**Affiliations:** 1 Max Delbrück Center for Molecular Medicine in the Helmholtz Association, Berlin, Germany; 2 German Center for Cardiovascular Research (DZHK), Berlin, Germany; 3 Charité Universitätsmedizin Berlin, Germany; 4 Berlin Institute of Health, Berlin, Germany; 5 Cardiovascular Research Center, Department of Internal Medicine, Yale University School of Medicine, New Haven, CT, USA; 6 Department of Molecular and Cellular Physiology, Yale University School of Medicine, New Haven, CT, USA; 7 PARCC, INSERM, Université de Paris, Paris, France

## Abstract

Hereditary hemorrhagic telangiectasia is an autosomal dominant disorder caused by mutations in the bone morphogenetic protein signaling pathway, leading to arteriovenous malformations. While previously thought to share molecular and cellular dysregulation, this study reveals highly distinct mechanisms depending on whether mutations occur in Alk1 or SMAD4. Loss of SMAD4 enhances endothelial cell responses to flow, including flow-regulated transcription and cell migration against blood flow, causing excessive pruning of capillaries and the formation of single large shunts. Conversely, Alk1 deficiency disrupts endothelial flow responses, including cell polarization and directional migration, leading to a dense vascular network and the persistence of a malformation nidus. In vivo cell population tracking of mutant cells validates unique endothelial cell migration defects. Mosaic cell culture models further illustrate that mutant cells co-opt wild-type cells driving distinct Alk1 or SMAD4 mutant-like behavioral defects. These findings demonstrate that arteriovenous malformations develop through fundamentally different cellular mechanisms based on the specific genetic mutation emphasizing the need for tailored diagnostic and therapeutic strategies.

## Introduction

The vascular system develops and remodels through a series of complex cellular processes that continuously adapt its shape and function to the local needs of tissues and organs^1–6^. Defects in the fine-tuned coordination of endothelial cell collective migration, proliferation, perfusion-mediated stabilization and cellular rearrangements in response to fluid shear stress can lead to vascular malformation that can severely threaten organ function, or cause bleedings and life-threatening complications. Such vascular malformations can be a consequence of sporadic somatic mutations in various genes, most notably related to oncogenic pathways, or caused by a combination of germline mutations and localized endothelial cell activation. Depending on where the mutations occur, the nature of the affected genes, and the hemodynamic influence of blood flow as well as inflammatory cytokines, these lesions can form in either high-flow or low-flow areas of the vascular tree. Understanding the precise molecular and cellular mechanisms that drive aberrant vessel formation is fundamental to the development of precision therapy.

Central to such understanding is the behavior of endothelial cells during formation and remodeling of the vascular network, as well as their interaction with surrounding pericytes and vascular smooth muscle cells. Exactly how the collective behavior of endothelial cells leads to the formation of a vascular network with hierarchical organization of arteries, capillaries and veins remains incompletely understood. Whereas the initial vasculogenic assembly of vessels^2,5^ and subsequent sprouting angiogenesis^3^ are largely independent of blood flow, the stabilization of nascent vessels and the orderly pruning process that leads to the mature network depend on both the biomechanical forces of blood flow and related biochemical signaling events^1,7–14^. Recent work identified that the orderly migratory response of endothelial cells to changing fluid shear stress - termed flow-migration coupling - drives functional remodeling. Failure of endothelial cells to engage in this flow-migration coupling process has been shown to occur in mouse models of Hereditary Hemorrhagic Telangiectasia (HHT), suggesting that this deficiency drives the formation of arteriovenous malformations in this disease ^7,9,10,15–18^.

HHT is a developmental vascular disorder ^19^. This germline heterozygous, autosomal dominant disease is characterized by development of shunts that connect arteries and veins that can occur in many organs (such as skin, nose, mouth and gastrointestinal tract mucosa, lungs and brain) and can be small (dilated small blood vessels called telangiectasias) or large (arteriovenous malformations, or AVMs)^19,20^.

The major genetic effectors causing this disorder are part of the Bone Morphogenetic Protein (BMP) signaling pathway ^21–24^. Loss of function mutations in the co-receptor Endoglin (encoded by ENG) ^25^, the type I receptor Alk1 (encoded by ACVRL1) and downstream DNA-binding SMAD4 (encoded by MADH4) account for the majority of diagnosed cases, and lead to HHT1^25^, HHT2^26^ or JP/HHT^22^, respectively.

When circulating BMP ligands bind to the extracellular domain of the Alk1-ENG heterotetramer, this facilitates a conformational change in the intracellular domains, subsequently leading to phosphorylation of receptor SMADs (rSMADs) 1, 5 and 9. These phosphorylated SMADs then bind to SMAD4 and translocate to the nucleus, where this complex regulates targeted gene expression. HHT develops in early adulthood, and as a homozygous loss-of-function in any of the effectors is embryonic lethal, accumulation of somatic mutations in the healthy allele drives the loss of function phenotype, in a process called loss of heterozygosity^23^. Shunts appear in a sporadic manner and do not affect the entire vascular network within a tissue, and different somatic mutations accumulate in different cells and vascular beds^27^. In order for these shunts to start developing in a mature and quiescent network, a “second hit” vascular injury, which causes a reactivation of the vascular bed and the exit from a quiescent state, is usually required to occur ^27,28^. Current treatment options include mostly symptomatic and surgically invasive approaches, and mechanism-based drug options are limited ^19,29^.

The unpredictable behavior, variable presentation, and limited therapeutic options of arteriovenous malformations (AVMs) pose significant clinical challenges, underscoring the need to elucidate the underlying molecular mechanisms. In our search for critical and common molecular mediators and cellular mechanisms of HHT, we find that loss of SMAD4 or Alk1 surprisingly shows very different effects and influences the endothelial response to fluid shear stress in an opposing manner, ultimately leading to different AVM pathomechanisms. Alk1 deficient cells show a delayed and reduced migration, coupled with dysregulation of cell cycle programs, causing AVMs to grow via hypo pruning and enlargement of capillaries, while loss of SMAD4 enhances collective migration against flow, coupled with dysregulation of cell fate programs, leading to hyper pruning of proximal capillaries and therefore precipitating a shunt. These findings provide valuable insights into distinct pathomechanisms that lead to AVM formation, offering potential avenues for improved diagnostics and mechanism based treatment options.

## Results

### RNA sequencing unveils distinct transcriptional regulation associated with Alk1 and SMAD4 loss of function

**Figure 1:**
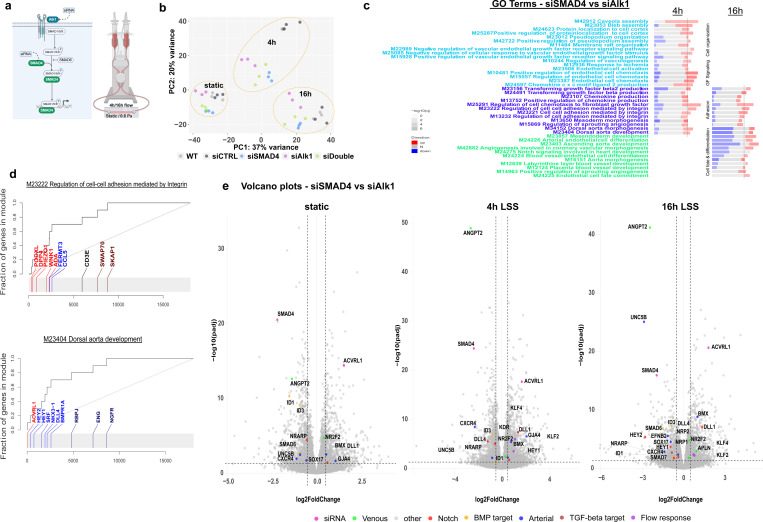
differential transcriptomic changes in ECs under flow a. Experimental schematic- HUVECs treated with siRNA against Alk1 or SMAD4 are exposed to 4 or 16 hours of laminar FSS 0.6 Pa (LSS hereafter). b. Principal component analysis of RNAseq samples. Dashed circles group samples by flow duration. n=3 independent experiments. c. Panel plot of select biological processes, significantly (adj. P-value<0.05) enriched in siSMAD4 treated samples vs siAlk1 treated samples after 4 and/or 16h of LSS. The length of the rectangle is the effect size (area under the curve or AUC, see Figure 1d). In red and blue are the fraction of significantly up- or down-regulated genes from that gene set. AUC_min_=0, AUC_max_=1. Vertical lines represent threshold value (AUC=0.65) for each column. d. Evidence plots of GO Terms representing differential regulation in two biological processes. X-axis is the list of all genes sorted by their p-value. Y-axis is the cumulative fraction. Light blue and light red colors represent significant down– or up-regulation. e. Volcano plots of differentially expressed genes in siSMAD4 treated samples vs siAlk1 treated samples, significance cut-off - Log2FC of at least 0.5. Select genes of interest are color-annotated. SMAD4 and ACVRL1 are annotated in pink to confirm knockdown.

In search for common pathogenic downstream effectors of Alk1 and SMAD4 loss-of-function we performed RNA sequencing on flow-mediated shear-stress exposed HUVECs following Alk1 or SMAD4 siRNA mediated knock-down (supplementary figure 1.1). The shear-stress magnitude was adjusted to 0.6 Pa to simulate shear-stress values typically occurring in veins and capillaries, as previous work has shown that AVMs in HHT models commence in capillaries and veins, but not in arteries^21^. RNA samples were collected after 4 and 16 hours of flow exposure (Figure 1a). Principal component analysis of all samples showed samples clustered primarily according to flow duration (Figure 1b), with untreated controls (light gray dots) and control siRNA treated samples (dark gray dots, siCTRL hereafter) clustering together. Surprisingly however, Alk1 siRNA and SMAD4 siRNA treated samples did not cluster together. This is remarkable, given that SMAD4 is a downstream component of the BMP9/10 signaling pathway triggered by the activation of Alk1. Instead, siAlk1 treated samples (magenta dots) clustered separately from siSMAD4 treated samples (blue dots), and samples treated with both siSMAD4 and siAlk1 (siDouble hereafter, green dots), clustered between the two single treatments at all time points. We therefore set out to investigate the differences in gene regulation in more detail.

Gene set enrichment analysis using GO biological processes (GO_BP hereafter) directly comparing siSMAD4 treated samples versus siAlk1 treated samples revealed 6 clusters, based on flow dependence and duration (Figure 1c and Supplementary Figure 1.2). Flow dependent GO_BP were divided into 3 clusters - differential expression at early flow exposure (light blue), flow-dependent differential expression (dark blue), and differential expression at late flow exposure (light green). Interestingly, the early and late flow exposure GO_BP were quite different, with early flow exposure showing an enrichment in upregulation of biological processes associated with cell compartment organization, chemotaxis, VEGF and TGF-beta signaling in siSMAD4 treated samples, whereas late flow exposure exhibited enrichment in downregulation of biological processes associated with cell fate and arterial differentiation in siSMAD4 treated samples, compared to siAlk1 treated samples.

Evidence plots and volcano plots from the early flow response as well as late flow response exposed many differentially expressed genes, suggesting Alk1 and SMAD4 loss of function leads to highly differential effects on the endothelial response to flow (Figure 1d–e and supplementary figure 1.3).

### Alk1 is crucial for flow-induced BMP pathway activation, while SMAD4 governs its duration

**Figure 2:**
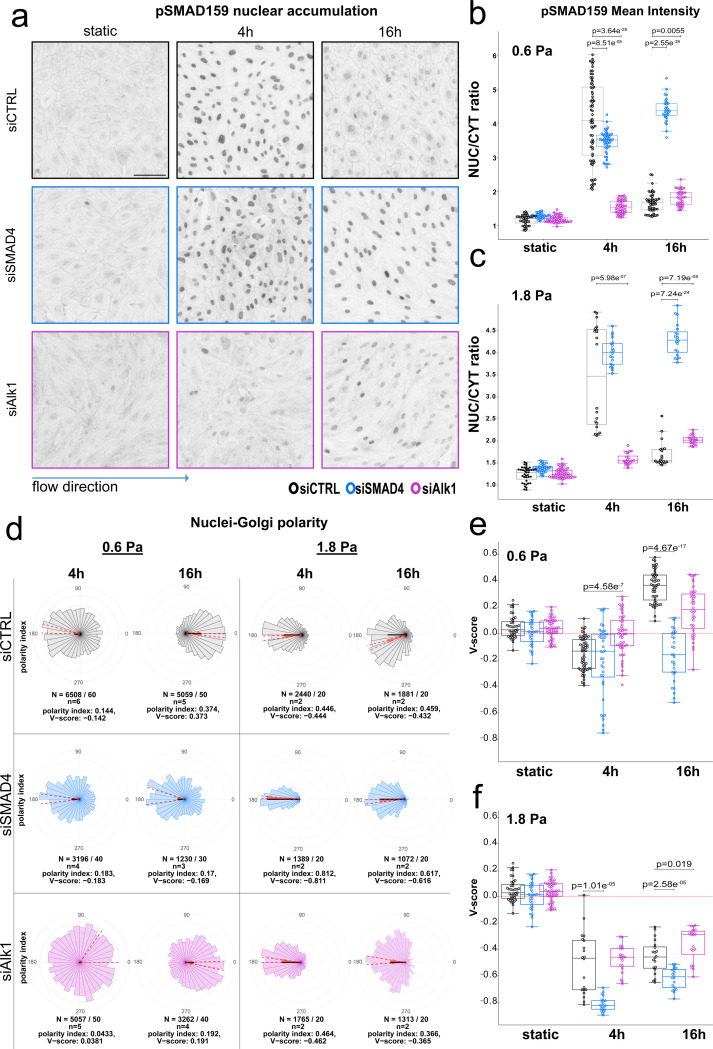
BMP pathway activation and cell polarity under flow a. pSMAD159 staining of HUVECs after exposure to LSS. Control cells have an initial activation of the BMP pathway which goes down over time, while siSMAD4 cells have constant BMP activity and siAlk1 cells don’t have BMP pathway activation. Scale bar 100μm. b. and c. Quantification of nuclear pSMAD159 signal, normalized to cytosolic signal at 0.6 and 1.8 Pa. Statistical analysis was performed using Welch’s test, n≥30 images per condition, from at least 3 independent experiments (b) and n=20 from 2 independent experiments (c). d. Rose diagrams depicting the Nuclei-Golgi polarity of siCTRL, siSMAD4 and siAlk1 treated ECs exposed to LSS or HSS (1.8 Pa) for 4 and 16 hours. The red arrow points towards the mean direction of polarization and its length represents the polarity index (PI), which is an indicator for the variance of the distribution; the black line represents the signed polarity index (V-score). Data underneath each rose diagram depict the total number of cells/images (N), number of experiments (n) and numerical values for PI and V-Score. e. and f. Graphical representation of V-score values from d for LSS and HSS (f), each data point representing mean V-Score of an individual image. Box plots display median values along with the standard deviation. Statistical analysis was performed using Welch’s test.

As SMAD4 is a direct transcriptional regulator of the Alk1-activated BMP pathway but also the TGF-beta pathway, it was essential to understand the roles of both Alk1 and SMAD4 on flow-induced BMP pathway activation. To test this, we exposed HUVECs to low shear stress of 0.6 Pa (LSS hereinafter) for 4 and 16 hours as in the RNAseq setup and assessed BMP pathway activation via the intensity of the nuclear pSMAD159 signal (Figure 2 a, b). In static conditions, HUVECs displayed basal nuclear pSMAD159 levels. Exposing the cells to 4h of LSS showed increased and variable activation of the BMP pathway, which however subsided after 16h in the siCTRL treated cells. The same flow regime in siSMAD4 treated cells led to a more uniform activation of the BMP pathway that remained active at 16h, while in siAlk1 treated cells no flow-induced activation of the BMP pathway was observed. Similar outputs were obtained for high shear stress levels (1.8 Pa, HSS hereinafter, Figure 2c and Supplementary Figure 2). Taken together, these data suggest an essential role for Alk1 in flow-induced activation of the BMP pathway, while SMAD4 absence results in a prolonged, more robust and uniform BMP pathway activation.

### SMAD4 and Alk1 deficiency differentially impact cell polarity in endothelial cells

Cell polarity is a crucial aspect of development that is often characterized by the asymmetric distribution of organelles in response to extracellular stimuli such as biophysical forces ^30^. Endothelial cells exhibit a striking ability to establish flow-directed polarity, characterized by the positioning of the Golgi apparatus against the direction of blood flow, during their migratory response^7,9,31^. There is growing evidence for changes in this front-rear polarity in AVMs, as well as in endothelial cells depleted of BMP pathway components ^21,24^. In order to characterize these changes, we analyzed the nuclei-Golgi polarity of HUVECs after exposure to LSS and HSS for 4 and 16 hours (Figure 2d–f). In LSS conditions, we observed a weak polarization of siCTRL treated ECs against the flow at 4h which reversed and intensified at 16h (Figure 2 d, e). Surprisingly, siSMAD4 treated cells polarized against flow direction in both time points, whereas siAlk1 treated cells exhibited random polarization. When exposed to HSS, cells polarized against flow in all treatments although with strongest effects in siSMAD4 treated samples (Figure 2 d, f). The fact that even siAlk1 treated cells polarize against flow, although they do not show induction of pSMAD1/5/9, suggests that polarization induced by HSS occurs independent of BMP pathway activation. In summary, our data shows that endothelial cells lacking SMAD4 or Alk1 respond very differently to fluid shear stress, particularly under LSS levels.

### Fluid-shear stress-dependent changes in cellular morphology are enhanced in the absence of SMAD4

**Figure 3:**
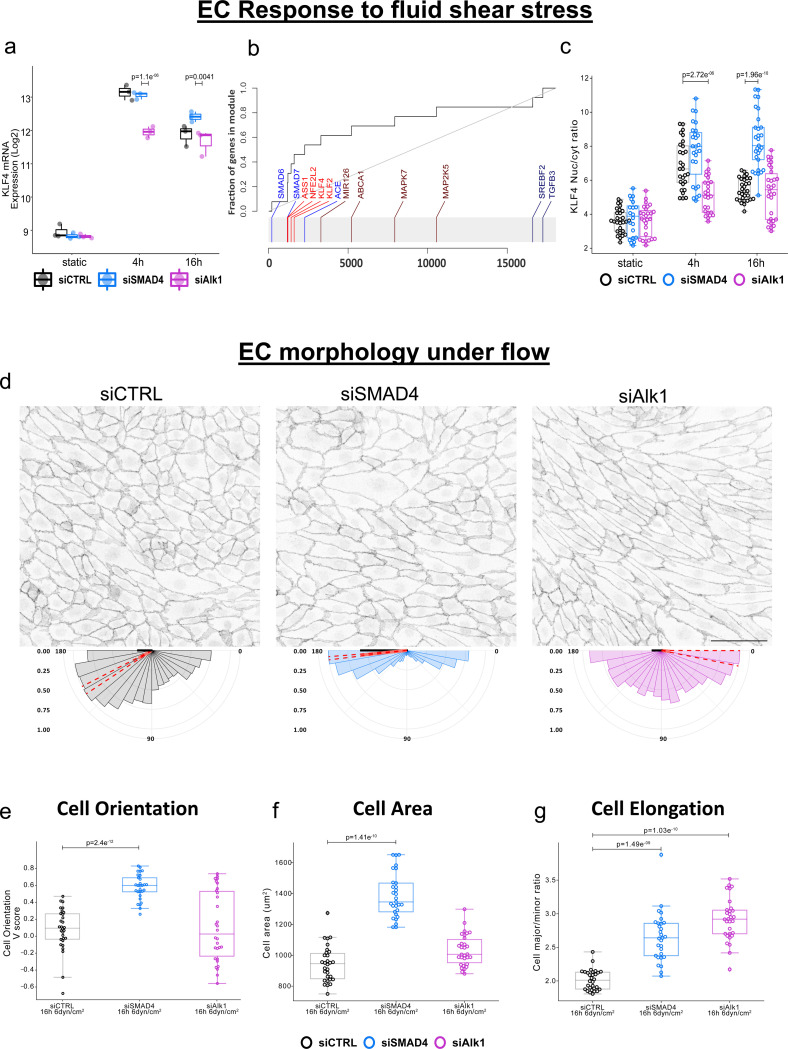
Endothelial response to FSS via KLF4 signaling and morphological changes a. Analysis of KLF4 mRNA expression after exposure to LSS in Log2FC scale. Static samples are relative to wild-type static, flow samples are relative to static samples for the same siRNA condition. siSMAD4 cells have higher upregulation of KLF4 while siAlk1 cells have a lower upregulation of KLF4, indicative of their disturbed flow sensation. Adjusted p-values (padj) are indicated. b. Evidence plot of biological process “response to fluid shear stress”, comparing siSMAD4 treated samples relative to siAlk1 treated samples after 16h of LSS. X-axis is the list of all genes sorted by their p-value. Y-axis is the cumulative fraction. Light blue and light red colors represent significant down– or up-regulation. c. Quantification of nuclear KLF4 signal, normalized to cytosolic signal. Statistical analysis was performed using the Welch’s test, n=40 images per condition, from 4 independent experiments. d. and e. Representative immunofluorescence staining of VEcadherin after 16h of LSS (top) and the corresponding cell orientation plots (bottom). e. Quantification of cell orientation V-score values. Box blots display median values and standard deviation. siSMAD4 cells orient themselves better parallel to flow than siCTRL cells, and siAlk1 cells form subpopulations that have different cell orientation. Statistical analysis was performed using Welch’s test, n=4 independent experiments with 10 images per experiment. Scale bar 100μm. f. Cell area analysis in μm^2^ and g. Cell elongation as ratio of major over minor axes of ECs after 16h of LSS. siSMAD4 cells are significantly bigger than siCTRL cells; siAlk1 cells are not bigger than siCTRL but are less variable in size and shape. Statistical analysis using Welch’s test, n=4 independent experiments with 10 images per experiment.

A key function of flow-migration coupling of ECs is the fine tuning of vessel diameter. This fine tuning depends on the ability of ECs to accurately sense flow and to undergo cellular rearrangements that affect their morphology, size and orientation. Our data demonstrate that siAlk1 cells fail to upregulate BMP signaling and to polarize against flow at lower shear stress levels, suggesting they might have either lost the ability to sense flow, or to respond to flow. The levels of Krüppel-like 4 (KLF4) transcription factor expression are established as flow sensitive^11,32,33^, and its mRNA levels increase in ECs upon exposure to flow. Analysis of KLF4 expression as surrogate for flow sensitivity confirmed that ECs sense the exposure to shear stress under the different siRNA knockdown conditions. siCTRL and siSMAD4 samples showed elevated KLF4 expression upon LSS exposure, and also siAlk1 samples showed elevated KLF4 expression, albeit to a significantly lower magnitude (Figure 3a). The notion that lack of Alk1 or SMAD4 differentially affects transcriptional response to flow is further supported by the evidence plot of the biological process “Response to fluid shear stress” (Figure 3b). Immunofluorescence staining for KLF4 protein also confirmed increased protein accumulation in the nucleus upon exposure to LSS. Albeit not to the same degree as seen at RNA level, siSMAD4 samples had elevated nuclear KLF4 accumulation in both time points (Figure 3c, Supplementary figure 3.1). In addition, we stained ECs for the adherens junction marker VE-Cadherin, allowing us to segment and analyze cell area, elongation and orientation and evaluate which of these are flow-dependent and/or LOF-dependent (Figure 3d–g, supplementary figure 3.2). Already in static conditions, siAlk1-treated endothelial cells in the monolayer formed distinct swirl-like clusters, where each sub-population maintained unique orientations independent of neighboring clusters. Despite their distinct alignment, these cell clusters remained in close proximity to each other, preserving their connections through adherens junctions (supplementary figure 3.2a). After 16h of LSS, the cell area of siAlk1-treated remained similar to siCTRL cells (Figure 3f, supplementary figure 3.2b). Although they were significantly more elongated (Figure 3g), the elongation of siAlk1-treated cells was not flow dependent (Supplementary figure 3.2c). In contrast, siSMAD4-treated cells had a significantly larger cell area (Figure 3f) compared to siCTRL and siAlk1. Additional analysis revealed that this enlargement was already significant prior to flow onset, but was enhanced by flow (supplementary figure 3.2b). siSMAD4-treated cells elongated and oriented themselves in a flow dependent manner (Figure 3e,g and supplementary figure 3.2c,d). Taken together, these observations further suggest an enhanced sensitivity to fluid shear stress in absence of SMAD4, and lowered sensitivity to fluid shear stress in the case of Alk1 absence.

### Endothelial cells exhibit distinct migration patterns in the absence of Alk1 or SMAD4

**Figure 4:**
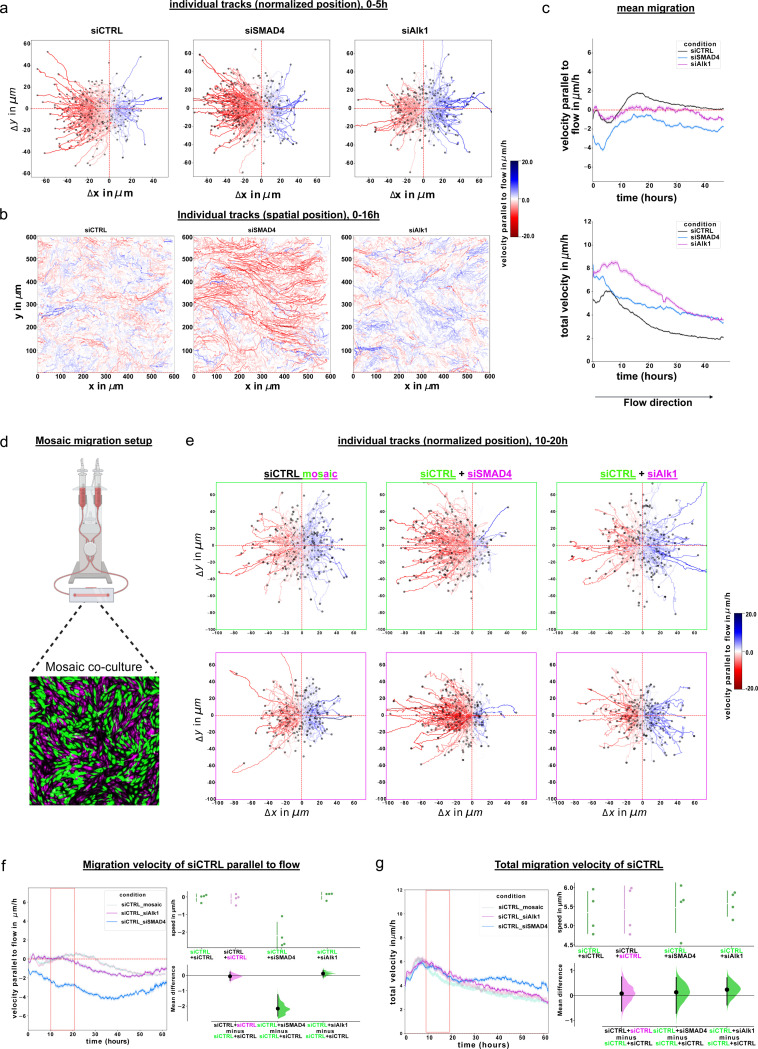
Endothelial migration in vitro a. Bootstrapped trajectory plots of initial 5 hours of migration for all conditions, 100 tracks per replicate, and 400 tracks per condition. Starting point of each cell trajectory has been normalized to start from (0, 0). Color intensity map represents scale of parallel migration velocity with flow (blue) to against flow (red). b. Representative track images from individual samples of the initial 16 hours of migration. Spatial position of each track is kept. c. Mean migration velocity of siCTRL, siSMAD4 or siAlk1 treated HUVECs, parallel to flow direction (top) or overall (bottom). n=4 individual experiments per siRNA treatment. d. Mosaic migration setup. Cells are separately labeled with CellTracker Green (green cells) or Red (magenta cells) and mixed before seeding in flow slides. e. Bootstrapped trajectory plots with normalized starting position of the green (top) and magenta (bottom) cells in each co-culture, from t=10 to t=20 hours. 100 tracks per replicate per color, from n=4 independent experiments. Color intensity map represents scale of migration velocity with (blue) flow to against (red) flow. f. and g. Mean migration velocity of siCTRL treated HUVECs when co-cultured either with themselves (siCTRL_mosaic, light pink/green curve), or with siSMAD4 or siAlk1 treated cells (siCTRL_siSMAD4, siCTRL_siAlk1, respectively), parallel to flow direction (f) or overall (g). Cells were labeled with CellTracker Green or Red (Thermofisher) and seeded together in 1:1 ratio. n=4 independent experiments. Red rectangles represent the time window of trajectory plot analysis. Statistical analysis: mean difference of the velocity parallel to flow (f) or total velocity (g), generated using estimation statistics. Both groups of siCTRL HUVECs migrate similarly when cultured together. siCTRL (green) HUVECs migrate at a similar speed when cultured with siAlk1 treated HUVECs, or faster parallel to flow when cultured with siSMAD4 treated HUVECs.

The end point analyses of SMAD4 and Alk1 deficient cells so far captured two distinct phenotypes that suggest hypersensitivity and hyposensitivity to LSS, respectively. We therefore hypothesized that siSMAD4 cells would migrate faster against flow, while siAlk1 cells would migrate less effectively against flow. By labeling the nuclei of ECs with Spy505 and tracking their movement over time under LSS conditions, we were able to analyze the migration pattern of cells in the different siRNA-treatment conditions (Figure 4a–c, supplementary movies 1a-c). Analysis of individual cell tracks as well as population behaviour revealed that control ECs exhibit a mild preference to migrate against flow (Figure 4a,b left panels, red tracks), with a smaller proportion of cells migrating with flow (blue tracks) or perpendicular to flow. siSMAD4-treated cells exhibited a much more prominent shift in trajectories against the flow direction and also less perpendicular trajectories. The fraction of cells migrating with flow was reduced, but surprisingly, they moved with higher velocities (Figure 4a, b middle panels). siAlk1-treated cells also showed an increased velocity as well as overall cell movement, but with the majority of cells migrating with flow. (Figure 4a, b right panels). When plotting the average migration of all cells parallel to flow for each condition, it became clear that control ECs initially migrate against flow, then reverse direction to move with flow, and ultimately slow down to cease migrating after 48h. siSMAD4-treated cells show a higher acceleration in net migration velocity against flow, but continue to migrate against flow throughout the duration of the experiment. siAlk1-treated cells initially behave similar to siCTRL-treated cells, but rapidly decrease net migration velocity parallel to flow (Figure 4c top panel). Intriguingly, siCTRL - treated cells show an average total velocity (irrespective of direction) that is slower than that of cells of the other conditions. siAlk1 - treated cells show the highest initial velocity and slow down over time to match the speed of siSMAD4 cells, but unlike the latter, they fail to migrate directionally against flow. These data strengthen the hypothesis that loss of SMAD4 renders ECs hypersensitivity to flow as they migrate more effectively against flow, while the loss of Alk1 leaves ECs unable to collectively respond to shear stress as they fail to migrate against flow, despite their high migratory potential. Control cells display a very characteristic biphasic response and strong flow adaptation, suggesting that the rearrangements of ECs as a consequence of shear stress represent a transitory adaptation mechanism.

As HHT progresses by the loss of heterozygosity via somatic mutations on the healthy alleles of BMP pathway components in a subset of endothelial cells within a vascular plexus, a model for cell migration that more closely resembles the in vivo situation of HHT would be to look at a mosaic scenario where only a subset of cells are deficient for SMAD4 or Alk1. By labeling the two populations with CellTracker dyes, we observed these mosaic scenarios under flow (Figure 4d–g, supplementary movies 2a-c). Intriguingly, in the mosaic situation, when siSMAD4 cells were cultured together with siCTRL cells, siCTRL cells migrated more effectively against flow (Figure 4e-middle panels and 4f-blue curve), however without elevation of their overall velocity, following the migration pattern of siSMAD4 cells (supplementary movie 2b, and figure 4g-blue curve). The opposite was observed when siCTRL cells were cultured with siAlk1 cells, as siCTRL cells exhibited a noticeable shift towards migrating with the direction of flow (Figure 4e right panel and 4f-pink curve). Interestingly, the average velocity of siCTRL cells increased later on, overcoming the initial delay (supplementary movie 2c and figure 4g-blue curve). Taken together, these findings suggest that the absence of SMAD4 not only results in cell-autonomous hypersensitivity of cells to FSS, but also that these cells affect control cells to migrate in a similar fashion. Likewise, the absence of Alk1 results in a cell-autonomous hyposensitivity of ECs to FSS, and a non-cell autonomous effect on the co-migration of control cells in the mosaic situation.

### Cells lacking Alk1 or SMAD4 exhibit distinct population movements in the retina in vivo

**Figure 5:**
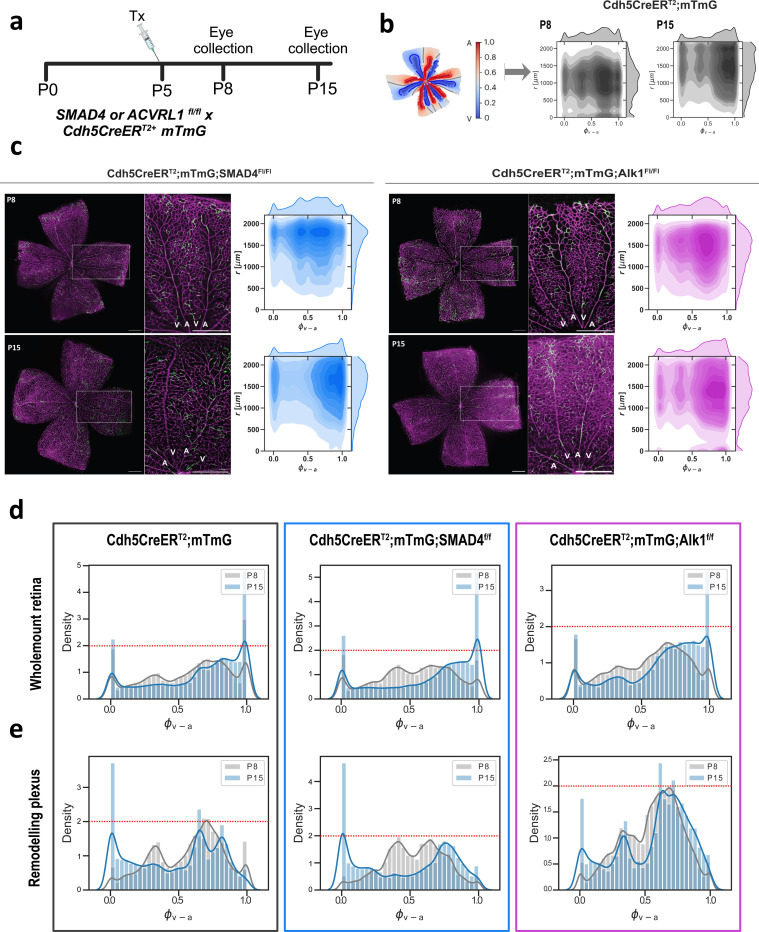
In vivo EC population distribution analysis a. Tamoxifen injection regimen. Single 0.75ug injection of T-OH at P5 to induce mosaic gene deletion and subsequent expression of GFP, collection at P8 and P15. b. Analysis overview: two-dimensional maps of fluorescently labeled ECs in flat-mounted retinas, result in a KDE plot, for which x axis depicts the relative distance between veins (0.0) and arteries (1.0); and y axis depicts radial distance (r[μm]) from optic nerve to sprouting front. KDE plots shown are for control retinas at P8 and P15. c. Representative images of Retinas at both time points for both SMAD4^mTmG^ and Alk1^mTmG^ conditions; dashed boxes are enlarged on the side with arteries (A) and veins (V) marked. Scale bar - 500μm. Resulting KDE plots for P8 and P15 for each condition on the right. d. and e. Density histograms on the arteriovenous axis depicting an overlay of P8 and P15 distribution of GFP+ ECs across the vasculature for CTRL^mTmG^, SMAD4^mTmG^ and Alk1^mTmG^ conditions, either across the whole mount retina (d) or within the remodeling plexus (e): 0.0 = vein; 1.0 = artery.

Should the loss of Alk1 or SMAD4 affect EC migration patterns in a similar way in vivo, we would predict that Alk1 knock-out ECs are impaired in their ability to move against flow, whereas SMAD4 knock-out cells migrate even more effectively than control cells. To assess this possibility, we turned to the mouse retina model, and employed mosaic endothelial Cre-lox mediated genetic labelling to follow population movements over time. Using our bespoke dual coordinate EC distribution analysis ^34,35^, we tracked the changes in cell population positions in mosaic retinae overtime. CDH5-Cre^ERT2^ mTmG (CTRL^mTmG^ hereinafter), CDH5-Cre^ERT2^ SMAD4^fl/fl^ mTmG (SMAD4^mTmG^ hereinafter) or CDH5-Cre^ERT2^ Alk1^fl/fl^ mTmG (ALK1^mTmG^ hereinafter) pups were treated with a low dose (0.75ug) of Tamoxifen at P5, to induce mosaic knockout of SMAD4 or Alk1, respectively. Retinae were collected at P8 and P15 and stained for GFP and IB4 (Figure 5a). The locations of GFP positive (GFP+) ECs were analyzed at both time points and their distribution illustrated in kernel-density estimation (KDE) plots (Figure 5b). Using this approach, we have previously shown that EC populations shift from the vein and plexus to the artery, as cells migrate against the direction of flow^34,35^. Whereas the GFP+ EC density within the artery was similar for control, SMAD4^mTmG^ and ALK1^mTmG^ retinae at P8 (supplementary figure 4a), the situation was very different at P15. Between P8 and P15 control retinas showed a prominent shift of cells to the artery (position 1.0 in the KDE plots, figure 5b and supplementary figure 4c). However, this was even more pronounced in the SMAD4^mTmG^ retinas (figure 5c, blue plots), but much less so in ALK1^mTmG^ retinas (figure 5c, pink plots; and 5d,e, compare levels indicated by red dash line). Further analysis of GFP+ ECs on the vein-artery axis confirmed a significant shift out of the venous bed for SMAD4^mTmG^ as well as a shift from the arterial bed into the arteries (supplementary figure 4c, blue dots), both when analyzing the whole retina (whole mount) and when considering only the remodelling plexus (Figure 5d,e middle panels). In contrast, ALK1^mTmG^ retinae did not exhibit a major shift within the venous and arterial beds both in whole mount and within the remodelling plexus (Figure 5d,e, right panels; supplementary figure 4c, pink dots). Statistical analysis of the arterial coordinates (90th percentile) confirmed a larger accumulation of GFP+ ECs within the artery in SMAD4^mTmG^ retinas (Supplementary figure 4d). Overall, these results demonstrate that the loss of SMAD4 or ALK1 in mosaic cells in vivo leads to differential migration defects, in line with our observations in the in vitro flow assays. Whereas the absence of SMAD4 promotes directional migration against flow, the absence of ALK1 impairs this migration from the veins to the arteries.

### Loss of SMAD4 leads to hyper-pruning, while loss of Alk1 causes hyper-sprouting during AVM formation in vivo

**Figure 6:**
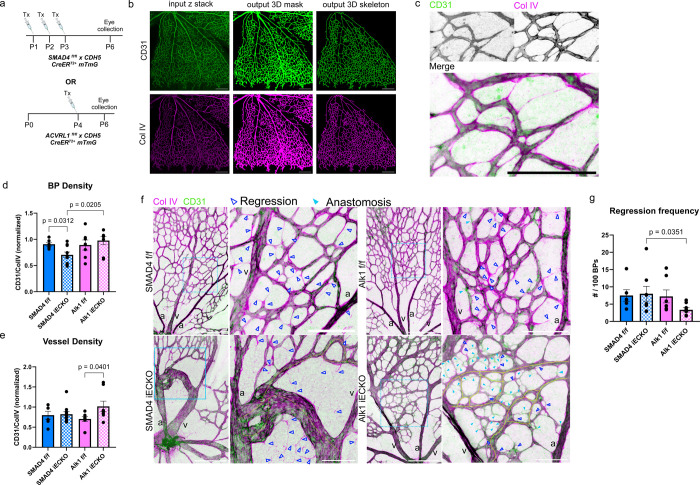
in vivo regression analysis in postnatal retina a. Tamoxifen injection regimen for SMAD4iECKO and Alk1iECKO, respectively. N_SMAD4 f/f_=6, N_SMAD4 iECKO_=8, N_Alk1 f/f_=7, N_Alk1 iECKO_=8. b. Representative maximum intensity projections of input z-stack, 3D mask and 3D skeleton output of CD31 channel (green, top row) and Col IV channel (magenta, bottom row). After segmentation of each channel and creation of a 3D mask, ROIs are selected for each channel. The tool creates a 3D skeleton of the selected ROI and outputs vessel density (vessel volume/ROI volume x100%), branching points density (# branching points/ROI volume), mean vessel length and mean vessel radius (μm), as well as absolute number of branching points and ROI volume (μm^3^). CD31/ColIV ratio is calculated for branching points’ density (e) and vessel density (f) and is normalized to the sample’s CD31/ColIV ROI volume ratio. Scale bar - 250 μm. c. Enlarged grayscale and merged channel images of Alk1 littermate control retina (b), depicting regression events. A regression event is classified by partial or total absence of CD31 signal within a vessel while ColIV signal is present. Scale bar 100 μm d. Branching points’ density and e. vessel density ratios in littermate controls (full-color bars) and KO retinae (dotted bars). Two-sided non-parametrical Mann Whitney test was used to determine statistical significance. Data are represented as mean ± SEM. f. Representative images of immunofluorescent staining of CD31 (green) and Collagen Type 4 (magenta) in P6 littermate controls (top) and mutated (bottom) retinae. Blue rectangles annotate zoomed in sections on the right. Dark blue unfilled triangles mark regression events, filled triangles mark anastomosis events. AVMs are highlighted in yellow dashed lines. Size marker 250μm in main images, 100μm in zoomed in images; v - vein, a - artery. g. Regression frequency, presented by number of regression events per 100 CD31+ branching points. Number of branching points is extracted from the 3DVascNet software for both CD31 and Col IV channels. Regression events are counted within a region of interest (ROI) which is also used to determine the number of branching points. Two-sided non-parametric Mann Whitney test was used to determine statistical significance. Data are represented as mean ± SEM.

Given that directional migration from vein to artery is the driving mechanism for pruning of the capillary plexus during vascular remodelling^36^, and that hypersensitivity to flow, or increased flow conditions cause hyperpruning^37^, we hypothesized that the loss of SMAD4 should trigger hyper-pruning, hence increased capillary regression. In contrast, reduced sensitivity and propensity to migrate against flow, as seen in ALK1 deficient ECs should rather trigger hypo-pruning, causing a hyperdense plexus. We therefore analyzed the regression frequencies in the postnatal retinal vasculature of both SMAD4 knockout (SMAD4^iECKO^ hereinafter) and Alk1 knockout (Alk1^iECKO^ hereinafter). Immunofluorescent labeling of CD31 and Collagen type 4 (ColIV) highlighted lumenized vessels and the basement membrane, respectively (Figure 6a–c). While SMAD4^iECKO^ retinas overall showed a similar regression frequency to its littermate control (Figure 6f - left, 6g), we observed a decrease in the branching points density (Figure 6d), indicating elevated vessel regression at earlier time points. In contrast, we observed a significant reduction in regression frequency in Alk1^iECKO^ retinae (Figure 6f right, 6g), together with increase in branching points density as well as vessel density (Figure 6d, e). In addition, Alk1^iECKO^ retinae exclusively exhibited an overall increase in sprouting frequency, which was also visible within the AVM region (Figure 6f, cyan triangles and Supplementary figure 5). Thus, SMAD4 deletion enhances remodelling of the vascular plexus, while Alk1 deletion delays it.

## Discussion

This study elucidates distinct molecular and mechanistic pathways underlying arteriovenous malformation (AVM) formation in endothelial cells (ECs) with SMAD4 and Alk1 loss-of-function (LOF). Our findings highlight that while both SMAD4 and Alk1 LOF contribute to AVM pathogenesis, the underlying cellular behaviors and transcriptomic responses differ markedly, pointing to divergent mechanisms driving these vascular pathologies.

The results reveal that SMAD4 LOF enhances EC sensitivity to fluid shear stress (FSS), leading to increased hypertrophy and directional migration. The observed increase in Klf4 expression upon SMAD4 deficiency confirms recent findings^24^, lending strength to the concept that AVM formation is primarily driven by changes in EC mechanotransduction^46^. The combined effects of increasing cell size, a phenotype first reported in ENG mutants in zebrafish^47^, and our unique observation of increased directional migration and polarization against flow, suggest an intriguing pathomechanism: This heightened migratory response accelerates vascular remodeling, resulting in premature capillary regression and the formation of large, high-flow AV shunts. Such a mechanism would explain the distinct appearance of AVMs in SMAD4 mutant retinas. Our data provide several levels of evidence for increased flow sensitivity in SMAD4 deficient ECs: First, transcriptional changes highlight besides classical flow-responsive genes like Klf4 and Klf2, also prominent flow-regulated upregulation of cell surface proteoglycans involved in endothelial permeability regulation and vessel lumen regulation, like PODXL^41–43,45^. Second, flow-mediated cell shape changes and directional polarity are surprisingly enhanced rather than diminished in the absence of SMAD4 (Figure 3). Third, directional EC population movements *in vivo* identify increased movement towards the artery, a behaviour depending on flow-responses^9^ (Figure 5). The cause for such enhanced flow sensitivity in SMAD4-deficient ECs can be manifold; our transcriptional analysis suggests disruptions in VEGFR2-mediated angiogenesis (Figure 1c, d and supplementary figure 1.3), driven by altered interactions between integrins and VEGFR signaling pathways. The increased expression of DPP4 and PODXL further modulates cell-ECM adhesion and migration^39,43^, likely contributing to the observed phenotypes. Intriguingly, SMAD4 LOF in the mouse retina leads to large shunts devoid of a surrounding capillary plexus. Yet, even the peripheral plexus that will be hypoxic, showing upregulation of hypoxia induced genes, like Angpt2^53^, shows no sign of hypersprouting, unlike what is the case in Alk1 mutant conditions. Thus, the lack of SMAD4 appears to selectively increase endothelial responses to flow, whilst reducing endothelial responses that normally drive sprouting. As EC responsiveness to FSS is ligand independent^69^, a mechanism by which ligand dependent VEGFR2 signaling is downregulated, thus tilting the signaling balance, can be explored.

In contrast, Alk1 LOF seems to diminish EC sensitivity to FSS, disrupting vascular remodeling and reducing capillary regression. We find reduced upregulation of Klf4, and reduced responses to FSS in all measured parameters, including reduced translocation of mutant cells *in vivo* (Figure 5). Such disrupted vascular remodelling results in hypervascularized networks with dense, multi-capillary AVMs (Figure 6d–f). Mechanistically, Alk1-deficient ECs fail to regulate vascular quiescence, including under flow, leading to hyperproliferation and impaired transitions from venous to arterial identity. These disruptions might involve altered BMP-Notch signaling crosstalk, as evidenced by flow-independent downregulation of venous markers (e.g., NR2F2, BMP4) and Notch-regulated arterial identity genes (Figure 1c–e and supplementary figure 1.3). Whereas previous studies comparing phenotypes and pathomechanisms of AVM formation between SMAD4, Alk1 and ENG mutants highlighted similarities, including upregulation of Angpt2^48^, we only find Angpt2 upregulation in Alk1 deficient cells, in line with the notion of failed induction of quiescence^49,50^. A possible reason for this apparent discrepancy is the fact that we analyzed cell autonomous gene regulation in ECs under flow, in the absence of pathology. Once an AVM has formed in vivo, studying altered gene regulation will report changes that may be primary, but also changes that are secondary to the altered tissue environment, including hypoxia. Angpt2 is a canonical marker of endothelial activation by hypoxia, but also in inflammatory conditions^51,52^. The fact that large areas of the endothelium upregulate Angpt2 in bulk RNA seq of isolated ECs from SMAD4 mutant retinas^53^, like those from Alk1 mutant retinas, is not necessarily evidence for a common causative Angpt2-induced pathomechanism. Nevertheless, Angpt2 overexpression may contribute as a driver of wider tissue responses in both conditions.

Our findings challenge the notion that AVM formation in SMAD4 and Alk1 LOF follows a unified pathway. Instead, they support a model in which SMAD4 LOF promotes FSS-driven vascular remodeling, while Alk1 LOF primarily disrupts cell fate acquisition and proliferation. These insights align with prior studies implicating BMP signaling dysregulation in AVM pathogenesis^26,54,55^, but also highlight the necessity of pathway-specific therapeutic strategies. For instance, targeting enhanced FSS sensitivity in SMAD4 LOF or restoring BMP-Notch crosstalk in Alk1 LOF could provide distinct avenues for intervention.

This study opens several avenues for future research. The observed differences in transcriptional and behavioral responses between SMAD4 and Alk1 LOF highlight the need for further mechanistic studies using multi-omics approaches. Combining transcriptomics, proteomics, and single-cell analyses across various tissue contexts could clarify which signaling effectors are primary drivers of AVM formation versus secondary responses to established pathology. Additionally, exploring factors such as ANGPT2 in Alk1 LOF and PODXL in SMAD4 LOF could uncover novel targets for modulating EC behavior.

Furthermore, the role of vascular permeability and immune-stromal interactions in AVM development remains underexplored. These factors could represent local “second-hit” mechanisms that exacerbate pathogenesis. Finally, sex-dependent differences^56^ in hereditary hemorrhagic telangiectasia (HHT) warrant further investigation, emphasizing the importance of precision medicine approaches.

This work reveals distinct, non-overlapping mechanisms of AVM formation in SMAD4 and Alk1 LOF. SMAD4 LOF enhances FSS sensitivity, driving premature remodeling and AV shunt formation, while Alk1 LOF disrupts quiescence and cell fate transitions, resulting in hypervascularized, dense AVMs. These findings provide a mechanistic framework for understanding AVM pathogenesis and underscore the need for pathway-specific therapeutic strategies in addressing vascular anomalies such as HHT.

## Methods

### Cell culture experiments

Human umbilical venous endothelial cells (HUVECs, PromoCell) were expanded and used in passages 2–4. Culture flasks were coated with 0.2% gelatin prior to seeding. Cells were cultured in EGM2-Bulletkit (Lonza) or MV2 (PromoCell) in a 37°C humidity incubator with 5% CO_2_. For siRNA knockdown, cells were seeded in T25 flasks and transfected after 24h at 60–70% confluence for siCTRL (Qiagen AllStars negative control, 40pmol), siSMAD4 (Qiagen, FlexiTube GeneSolution for SMAD4: SI03089527, SI03042508, SI00076041 and SI00076020; 10pmol each) or siAlk1 (Qiagen, FlexiTube GeneSolution for Alk1: SI02659972 and SI02758392, 10pmol each), using RNAiMax transfection reagent (Thermofisher) diluted in OptiMem media and added to antibiotic free EBM2 media (Lonza). After 5h, transfection mix was aspirated and replaced with full media.

### End point flow experiments

HUVECs were harvested 24h after siRNA knockdown and seeded onto gelatinized 0.4 Luer ibiTreat slides (Ibidi) at a concentration of 2 million cells per ml and placed overnight in a CO_2_ incubator. The following day, slides were connected to perfusion units (Ibidi) and exposed to 0.6 Pa or 1.8 Pa shear stress for 4h. Cells that were exposed to shear stress for 16h would be connected later on, and would have a media exchange earlier that day. A static control for each condition was placed in the flow incubator for the same duration as the flow experiments. After each flow experiment, cells would either be fixated with 4% PFA for subsequent immunofluorescence assays, or lysed with RLT buffer (RNAeasy RNA extraction kit, Qiagen) for RNA based subsequent analysis.

### RNA extraction for qPCR, RNA seq

After flow experiments, cells were lysed with two rounds of 200ul RLT buffer and collected into tubes. Cell lysates were kept in −80°C freezers until all relevant samples have been collected, then RNA extraction would be completed with the RNAeasy kit according to manufacturer’s instructions, including DNAse step. RNA concentration was assessed using NanoDrop. RNA was sent to the genomic core facility for RNA sequencing, or used for qPCR analysis. cDNA synthesis was carried out using BioRad’s or Thermofisher’s cDNA kits according to manufacturer’s instructions. qPCR was performed using Taqman probes on a QuantStudio 6 qPCR machine in 384 well plates.

### Bioinformatic analysis

RNA-Seq reads were mapped to the human genome (GRCh38.p7) using STAR (version 2.7.3a^57^). The reads were assigned to genes with FeatureCounts (version 2.0.3^58^) using Gencode version 25 (Ensembl 85) annotation and the following parameters “ -t exon -g gene_id -s 2 -p”. Differential expression analysis was carried out using DESeq2 (version 1.38^59^) using default parameters. Only genes with at least 5 reads in at least 3 samples were considered for the analysis. Gene set enrichment analysis was carried out with CERNO test from the R tmod package (version 0.50.13^60^) using MsigDB Biological Pathways GO collection.

### Immunofluorescence, imaging of *in vitro* flow samples

After fixation, slides were blocked and stained with primary antibodies against VEcadherin (goat, AF938, R&D Systems, 1:1000); GM130 (mouse, 610822, BD Bioscience, 1:500); pSMAD159 (rabbit, 13820S, Cell Signaling, 1:500) or KLF4 (rabbit, HPA002926, Sigma Aldrich, 1:500). Secondary antibodies were used in 1:400 dilution: Donkey anti goat IgG 568 (A11077, Thermofisher); Donkey anti mouse IgG 488 (A21202, Thermofisher); Donkey anti rabbit IgG 647 (A31573, Thermofisher). Nuclei were stained using a 1:1000 DAPI solution. Mowiol mounting media was mixed with Dabco solution (Sigma) at 10:1 ratio and applied to slides.

Slides were imaged on the Zeiss 980 Confocal inverted microscope with a 20x air objective by taking 10 non overlapping z-stack images per slide.

### Image analysis of *in vitro* data

Images were processed using sum intensity projection and were then analyzed using the Polarity-JaM toolbox and web-application^61^. The full code is available at https://github.com/polarityjam. Polarity analysis (for nuclei-Golgi polarity, cell orientation) was performed via acquisition of a polarity index (PI), which is an indicator of the concentration of the circular distribution, its value (limited between 0 and 1) indicating the collective orientation strength of the monolayer^62^. In addition, the signed polarity index (V-score) indicates the strength of polarization with respect to a given direction. The signed polarity index varies between −1 and 1 and indicates the strength of polarization with respect to an assumed direction of polarization.

Additional features were extracted through the web application www.polarityjam.com:

Nuclear marker accumulation was analyzed via the ratio of the mean intensity of pSMAD159/KLF4 in the nucleus and cytosol (NUC/Cyt ratio). Cell area was extracted and normalized to μm^2^. Cell elongation was extracted as the major/minor axis ratio. Circular statistics was performed via the PolarityJaM web application. Cell orientation of each condition was extracted as V-score per image from the circular statistics file.

### Live migration under flow

For live migration experiments, cells were prepared and seeded in the same conditions as described for the end-point flow experiments. On the day of the experiment, seeding MV2 media was aspirated and replaced with CO2 independent media (Promocell basal media without phenol-red and without sodium bicarbonate, supplemented with MV2 supplement kit, B-glycerolphosphate at a final concentration of 4.32ug/ul and sodium bicarbonate at a final concentration of 0.0075%) with addition of Spy505 (Spyrochrome) nuclear dye at 1:1000 dilution and incubated at 37°C for 4h. Fluidic units would be placed inside the incubation chamber of the microscope and warmed up prior to connection of slides. Slides were connected and placed on multi slide stage insert. Time Lapse imaging would run for 48h and acquire 3×3 tiles with 3 slice Z-stacks for each position, with a time interval of 7.5 minutes. Migration analysis would then be done using the TrackMate feature^63^ on Fiji (ImageJ) with StarDist segmentation tool^64^. Tracks would then be analyzed using a Python based script^65^ on Jupyter notebook (AnaConda).

For mosaic migration experiments, prior to seeding into flow slides, cells were labeled with CellTracker Green or Red (Thermofisher) with 1:5000 dilution (final concentration 2nM) in EBM2 media and incubated for 45 minutes in 37°C, then washed three times with full MV2 media and placed in the incubator for about 4h. Cells were then trypsinized and harvested, and seeded in the same concentration with 1:1 ratio between green and red cells. Live imaging would begin the following day, 48h after siRNA treatment. Time Lapse imaging was configured in the same manner, and ran for approx. 2.5 days (500 cycles with 7.5 minute intervals). Subsequent analysis for each channel was done in the same manner as described in the previous paragraph.

### In vivo experiments

All animal experiments were performed under a protocol approved by the Institutional Animal Care Use Committee of Yale University (no.2023–11406).

Seven to eight weeks old Alk1^f/f^ or SMAD4 ^f/f^ mice with Cdh5 CreERT2 and mTmG mixed genetic background were intercrossed for experiments and Alk1^f/f^ Cdh5 CreERT2 (mTmG) mice or SMAD4 ^f/f^ Cdh5 CreERT2 (mTmG) were used. Gene deletion was induced by intra-gastric injections with 100 μg Tamoxifen (Sigma, T5648; 2.5 mg ml−1) into pups at P4 or P1–3 to stain Collagen type 4 and CD31, and 0.75 μg Tamoxifen into P5 pups for mosaic knockout and labeling. Tx-injected CreERT2 negative littermates were used as controls. Retinas were collected on P6 for Collagen type 4 and CD31 staining, and on P8 and P15 for mosaic population analysis.

### Immunostaining and imaging of mouse retinas

Retinas were prefixed in 4% PFA for 8 min at room temperature. Retinas were dissected, blocked for 30 min at room temperature in blocking buffer (1% fetal bovine serum, 3% BSA, 0.5% Triton X-100, 0.01% Na deoxycholate, 0.02% Sodium Azide in PBS at pH 7.4) and then incubated with specific antibodies in blocking buffer overnight at 4C. The next day, retinas were washed and incubated with IB4 together with the corresponding secondary antibody overnight at 4°C, then washed and post-fixed with 0.1% PFA and mounted in fluorescent mounting medium (DAKO, USA). High-resolution pictures were acquired using a Leica SP8 confocal microscope with a Leica spectral detection system (Leica TCS SP8 detector), and the Leica application suite advanced fluorescence software. Primary antibodies: IB4 ([IsolectinB4] #132450, 1:400; Life Technologies), GFP Polyclonal Antibody, Alexa Fluor 488 (#A-21311, 1:1000; Invitrogen), CD31 (553370; 1:200; BD), Collagen IV antibody (2150–1470; 1:400; Bio-Rad).Secondary antibodies: Alexa Fluor 488 anti-Rat (A21208, 1:500, Invitrogen), Alexa Fluor 568 anti-Rabbit (A10042, 1:500, Invitrogen).

### Image analysis of in vivo data

Analysis of mosaic labeled mouse retinae was done as previously described^34,35^. Briefly, maximum projections of the IB4 channel were used to create drawn masks of retina outline, veins, arteries and optic nerve in Fiji^66^. Maximum projections of the GFP channel were used to create GFP masks.

For every pixel in the GFP mask, three numbers were computed (using drawn masks as referential): (1) distance to the nearest vein (d_v_); (2) distance to the nearest artery (d_a_); and (3) radial distance to the optic nerve (d_r_). From these measures, the relative distances by ϕ_v-a_ = d_v_/(d_v_ + da) were obtained. The EC distribution was computed by performing the operation for x randomly selected GFP-positive pixels in each retina, which were used as a proxy for EC distribution. A kernel density estimation was used to approximate the underlying EC distribution in the two-dimensional coordinate system spanned by ϕ_v-a_ and d_r_. For computational analysis, a Python-based workflow was used, accessible on GitHub https://github.com/gerhardt-lab/retina-VACS-HHT.

Analysis of mouse retinae used in regression analysis was done using the 3DVascNet software^67^. Briefly, z-stacks of CD31 and ColIV channels were uploaded into the software, along with a resolution file depicting the micron/pixel ratio of each image. Channels of the same retina were segmented independently from each other. After segmentation, a region of interest (ROI) was selected for each image, generally encompassing one retina leaflet. ROIs are skeletonized in 3D using the generated 3D mask from the segmentation step. The software outputs feature quantifications, including ROI volume and number of branching points. Regression events for each retina are quantified using a composite of the CD31 and ColIV channels and within the borders of the analyzed ROI. A regression event is classified by an absence of the CD31 signal, while ColIV staining remains intact. Partial absence of CD31 indicates the vessel is in an intermediate regression stage; complete absence of CD31 while ColIV staining remains intact indicates the final regression stage of a vessel; complete absence of CD31 and partial disruption of ColIV staining indicates long regressed vasculature. Regression frequency is quantified via the proportion of regression events per 100 branching points in the CD31 channel. Branching points and Vessel density ratios are quantified by dividing the outputs of the CD31 by ColIV channels, and subsequent normalization to the ROI volume ratio.

### Statistical Analysis - DABEST method

In order to compute effect sizes along with common p-value statistics, we used the DABEST (‘data analysis with bootstrap-coupled estimation’) method^68^. The mean difference distributions including 95% confidence intervals were plotted to indicate effect size. Common statistical analysis include two-sided Welch’s test, student t-Test and a non-parametric Mann-Whitney test. The test used is indicated in the figure captions.

## Supplementary Material

Supplement 1

Supplement 2

Supplement 3

## Data Availability

The raw RNA-Seq reads are deposited in the GEO database under accession number GSE282952.
