# Supplementary material for: Divergent endothelial mechanisms drive arteriovenous malformations in Alk1 and SMAD4 loss-of-function
Source: bioRxiv. 2025 Jan 3:2025.01.03.631070. Preprint. [Version 1] doi: 10.1101/2025.01.03.631070 (PMC11741317; doi:10.1101/2025.01.03.631070)
Supplement: Supplement 1 [file media-1.pdf]

Supplementary figure 1.1

Knockdown verification

qPCR

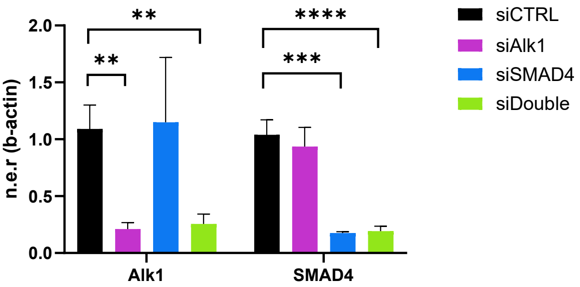

Western blot

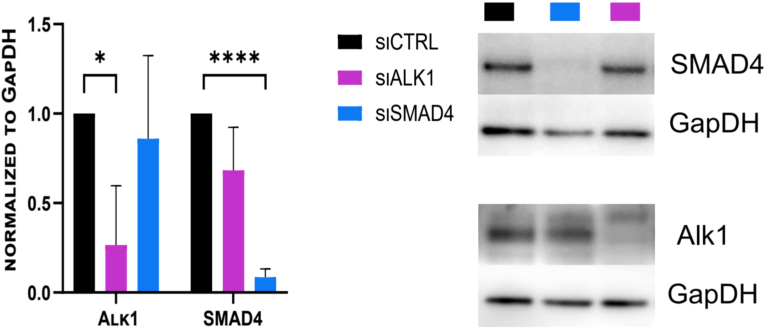

Supplementary figure 1.2

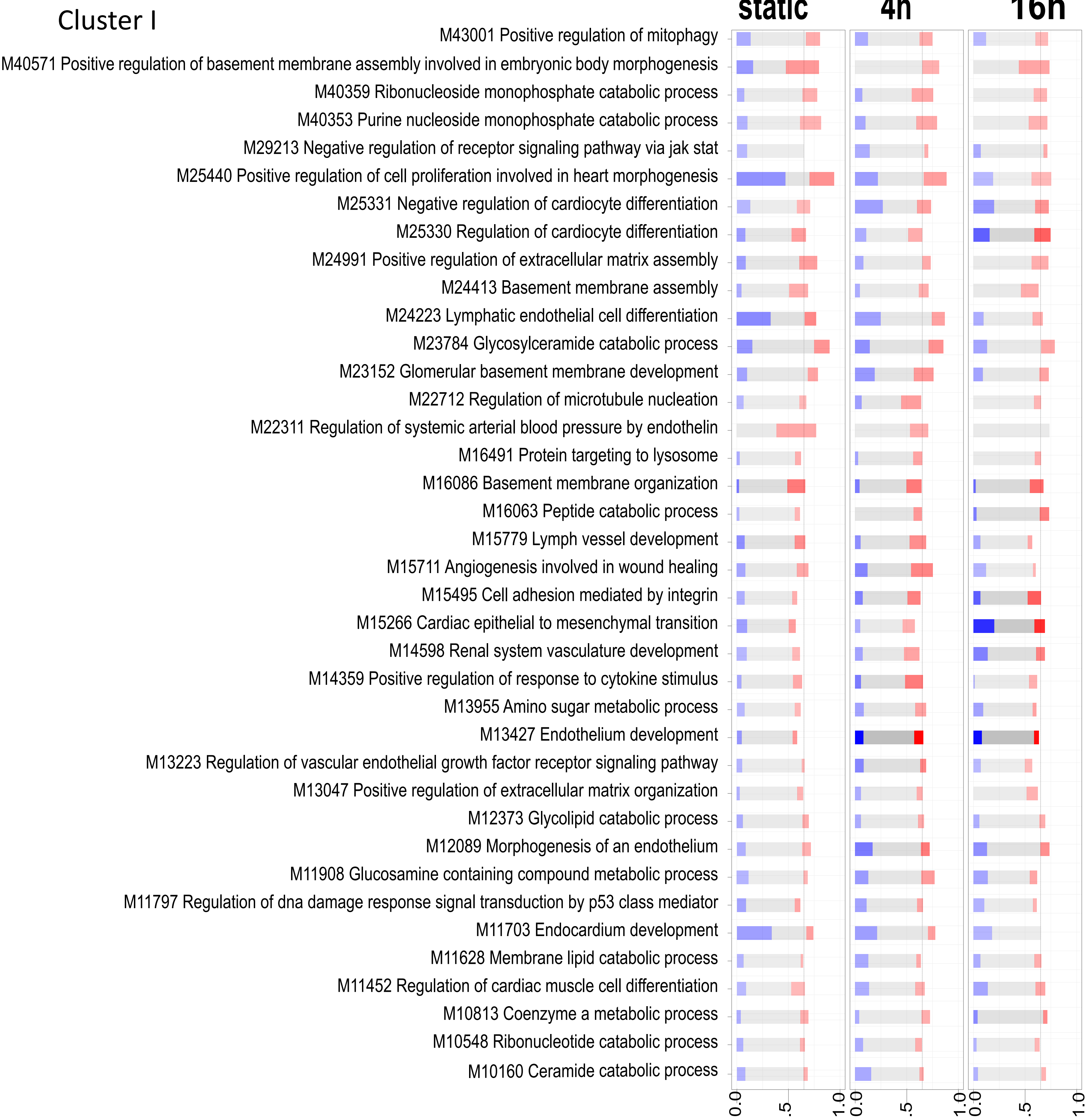

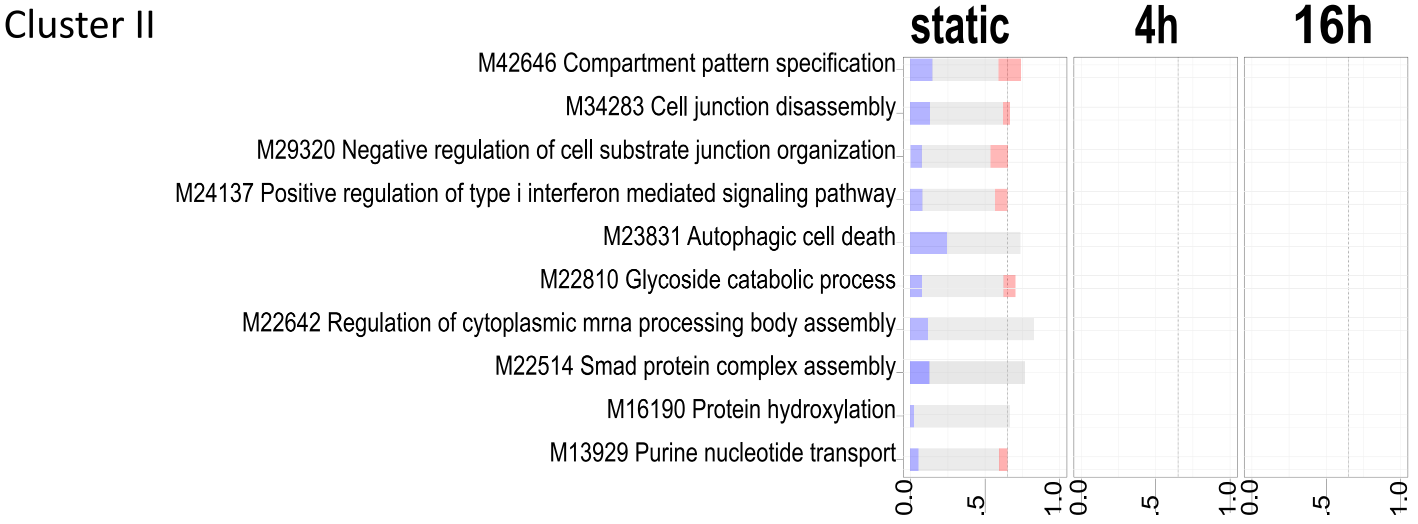

Supplementary figure 1.2 - continued

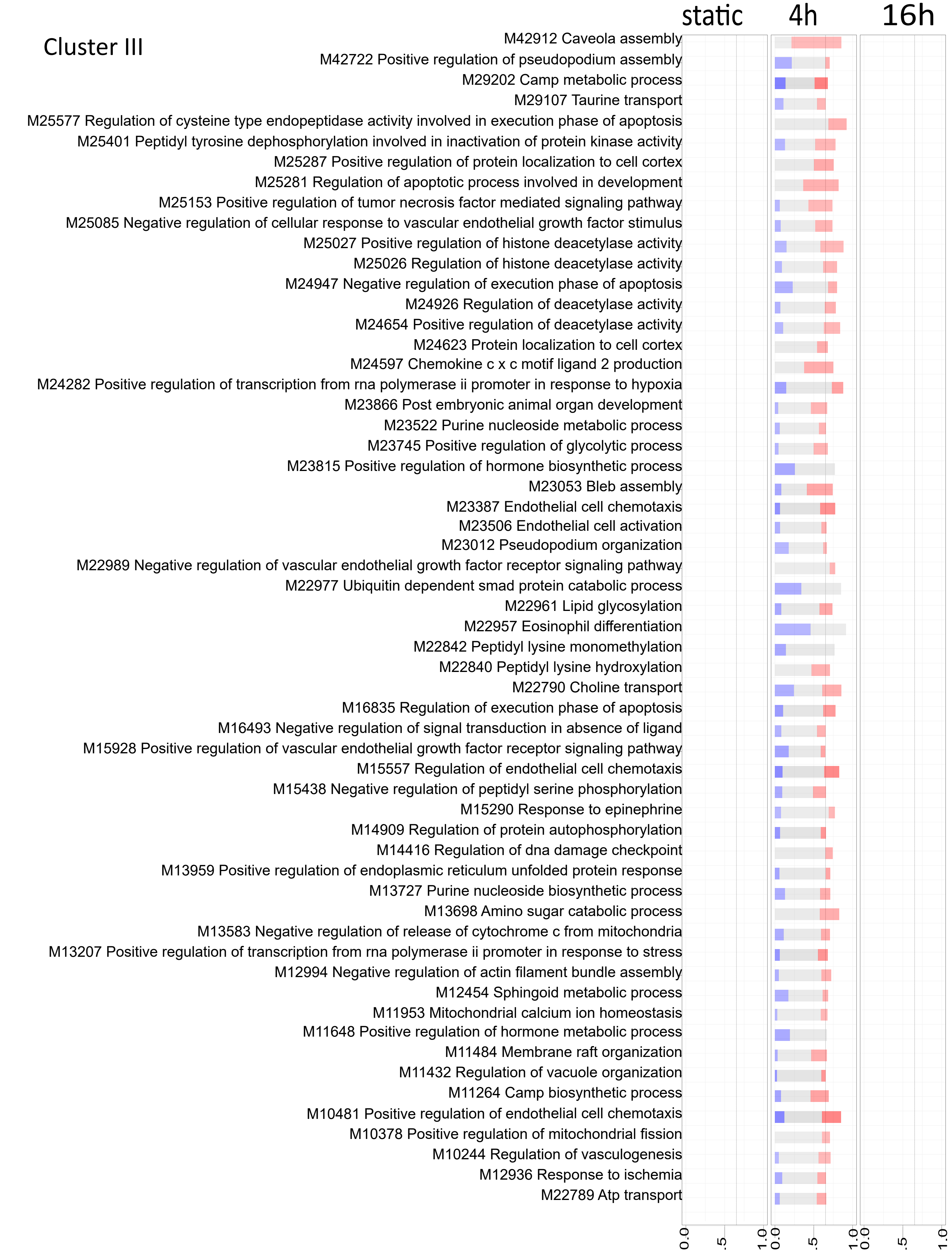

Supplementary figure 1.2 - continued

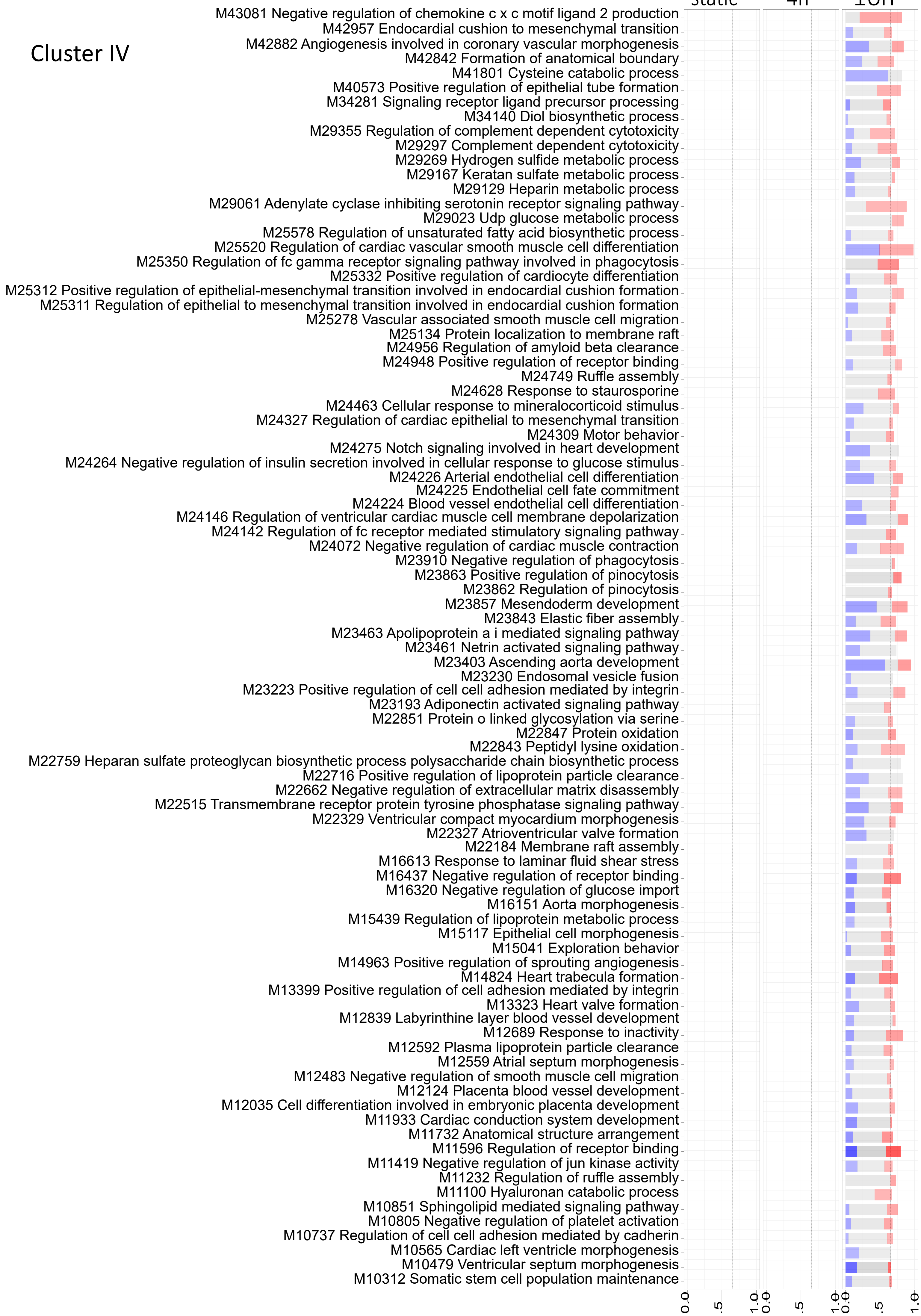

Supplementary figure 1.2 - continued

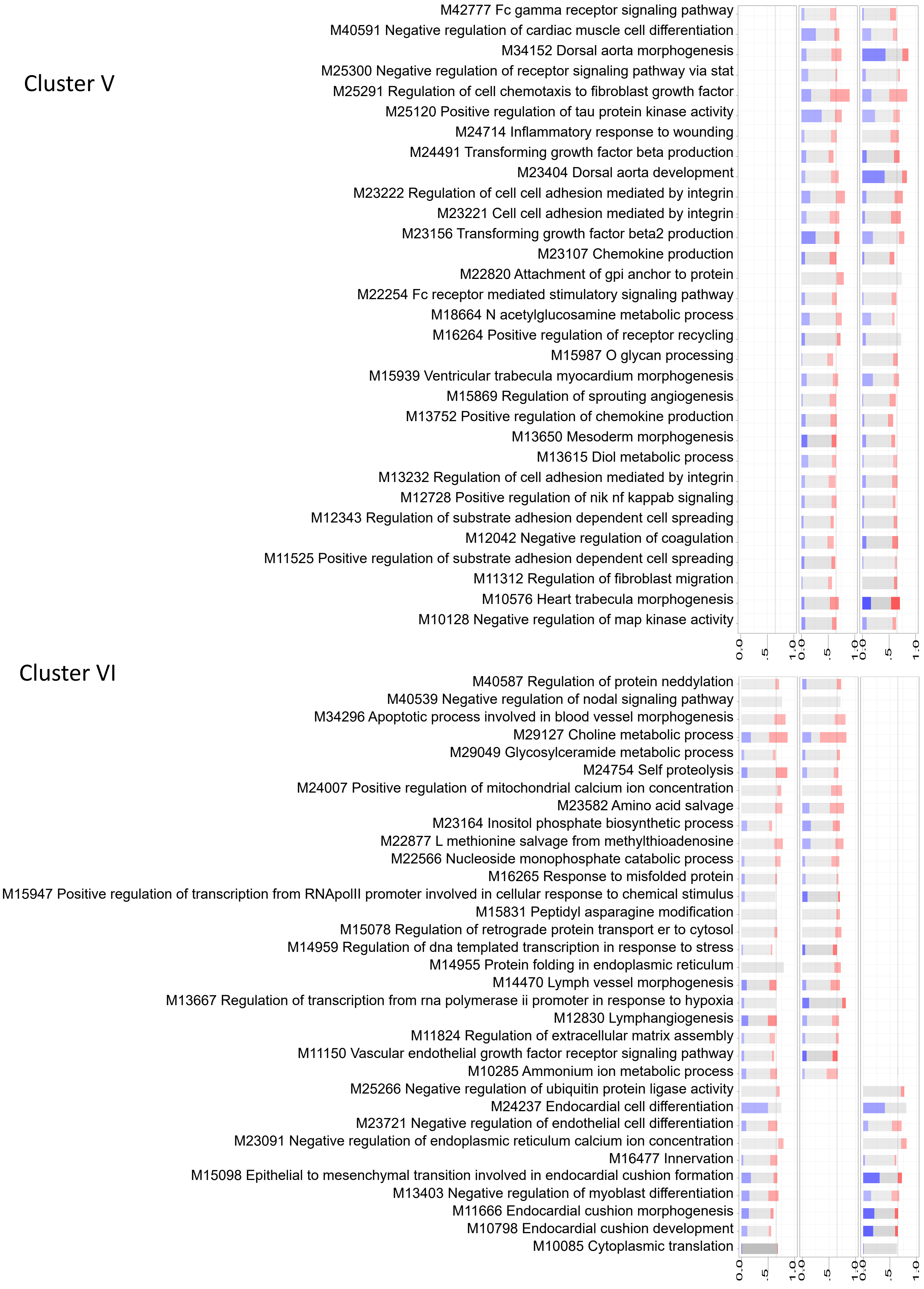

Supplementary figure 1.3

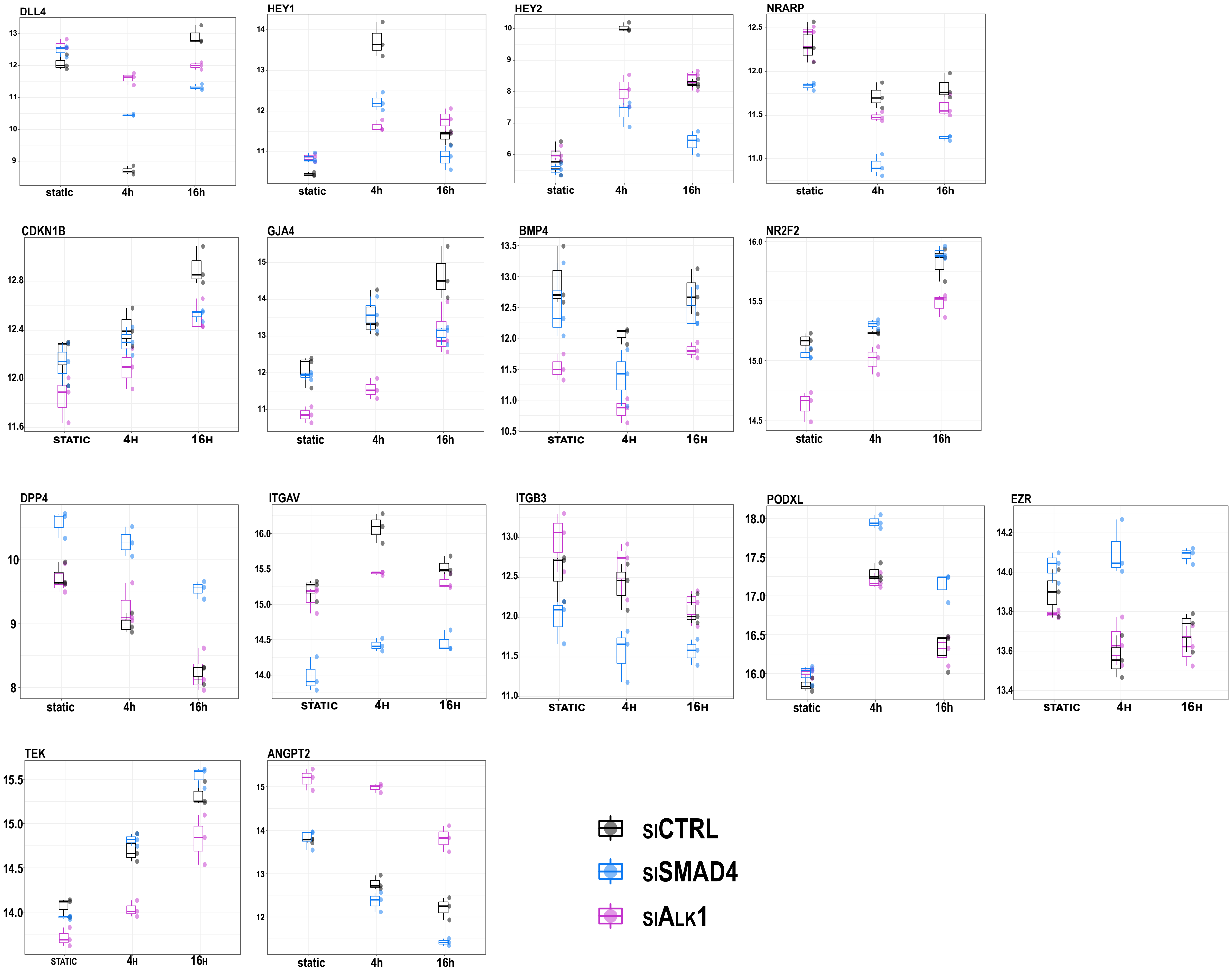

Supplementary figure 1:

1.1 Knockdown verification of SMAD4, Alk1. Top – qPCR analysis of siCTRL (black), siSMAD4 (blue), siAlk1 (magenta), siDouble (lime), 48h post siRNA treatment. Log2FC values represent relative expression to b-actin. N=3 independent experiments. Bottom – Western blot analysis of siCTRL, siSMAD4 and siAlk1 with representative images of blots, 48h post siRNA treatment. Bands normalized to GapDH; n=3 independent experiments.

1.2 Full panel plot of GO_BP of siSMAD4 vs. siAlk1 in static, 4h and 16h of LSS. GO_BPs are clustered according to their enrichment in the different flow conditions: Cluster I – enrichment in all flow conditions; Cluster II – enrichment in static only; Cluster III – enrichment in 4h LSS only; Cluster IV – enrichment in 16h LSS only; Cluster V – enrichment in both 4h and 16h LSS; Cluster VI – enrichment in static and a 4h LSS **OR** 16h LSS.

1.3 Expression patterns of individual genes over time (RNAseq data) first row: expression patterns of DLL4, HEY1, HEY2, and NRARP. Second row: Expression patterns of CDKN1B (p27), GJA4 (Cx37), BMP4 and NR2F2. Third row: Expression patterns of DPP4, ITGAV, ITGB3, PODXL and EZR. Fourth row: Expression patterns of TEK (Tie2) and ANGPT2.
SiCTRL – black; siSMAD4 – blue; siAlk1 – magenta.

Supplementary figure 2

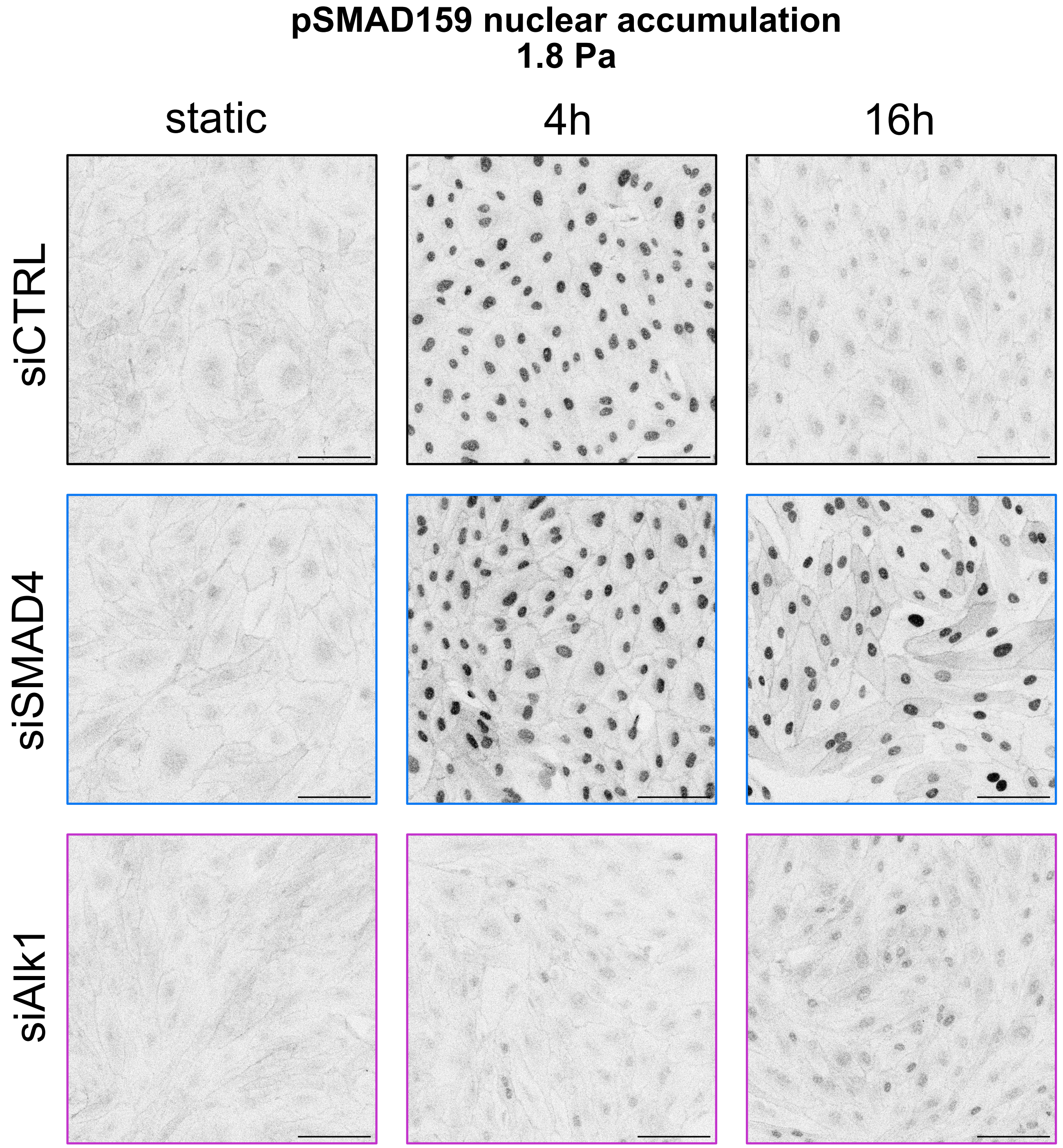

Supplementary figure 2:

Representative pSMAD159 immunofluorescence images of HUVECs after exposure to HSS (1.8Pa). Scale bar 100μm.

Supplementary figure 3.1

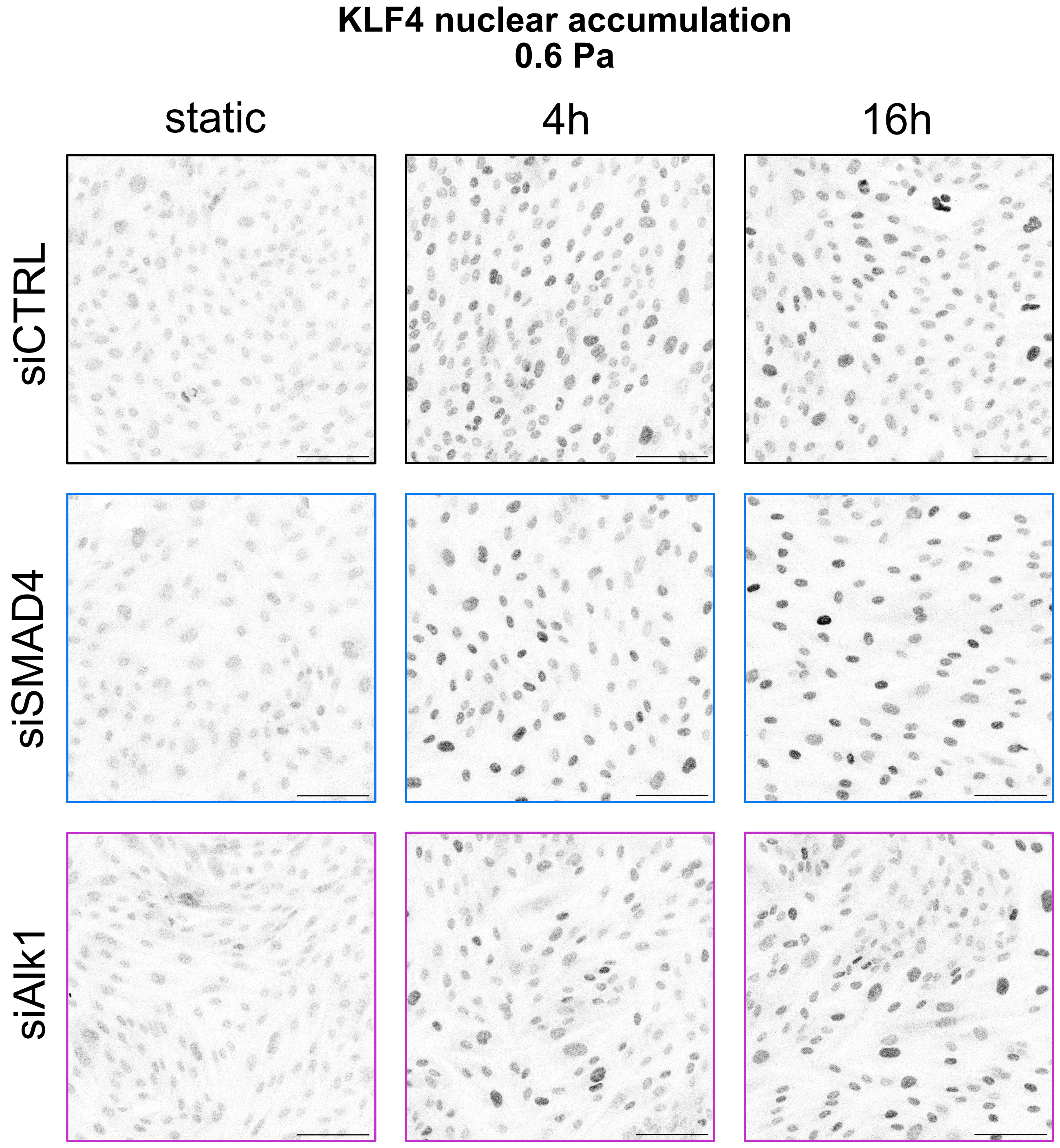

Supplementary figure 3.2

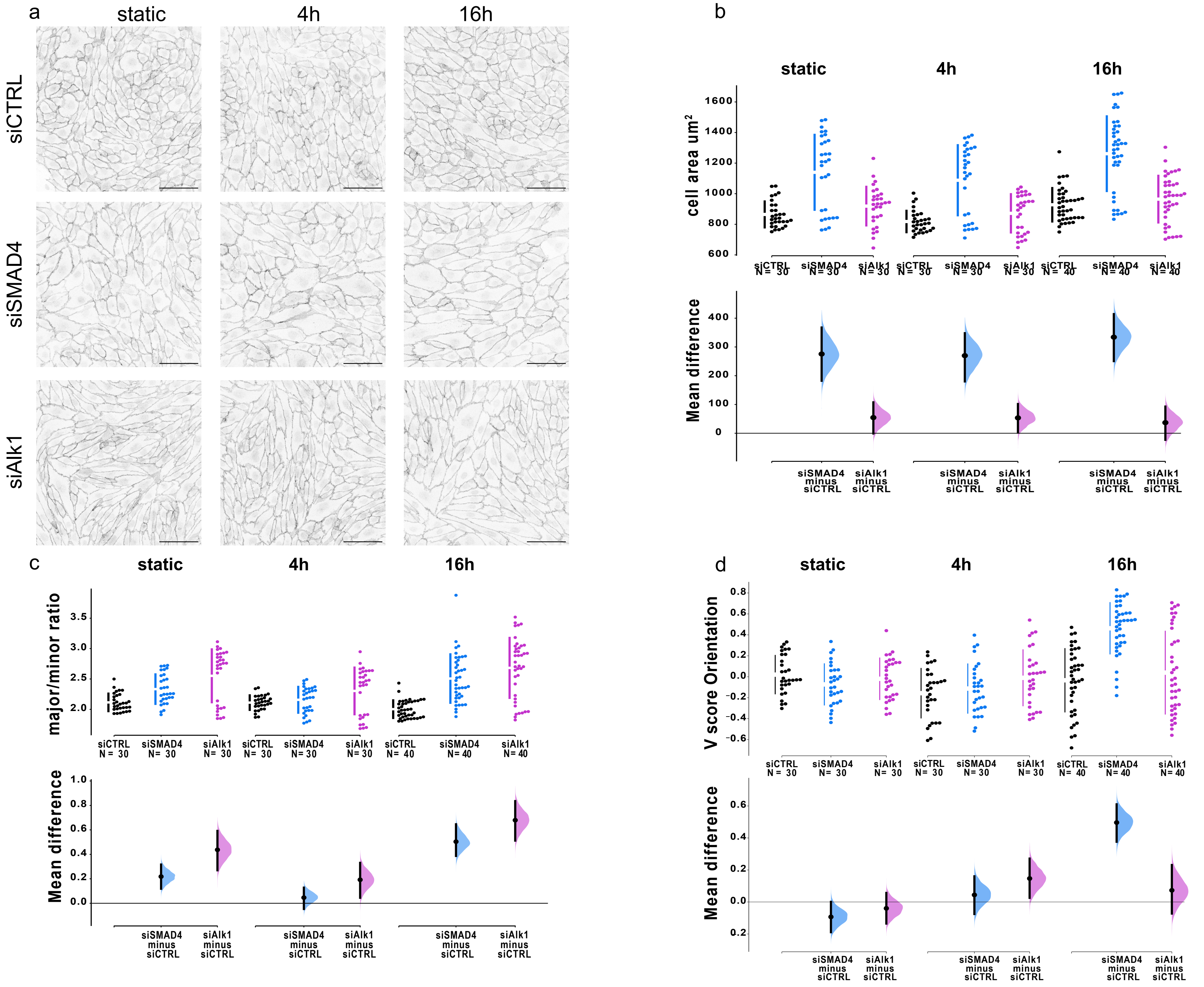

Supplementary figure 3:

3.1 Representative KLF4 immunofluorescence images of HUVECs after exposure to static, 4h and 16h LSS conditions. Scale bar 100µm.

3.2 Cell shape analysis over time. a. VEcad staining of HUVECs after exposure to static, 4 or 16h of LSS (0.6 Pa). Scale bar 100µm. b-d. Mean difference statistical analysis of cell area (b), cell elongation (c) and cell orientation (d) of siCTRL, siSMAD4 and siAlk1 treated ECs at static 4 and 16h of LSS. The further away the difference histogram is from the control group line (0.0), the higher the significance.

Supplementary figure 4

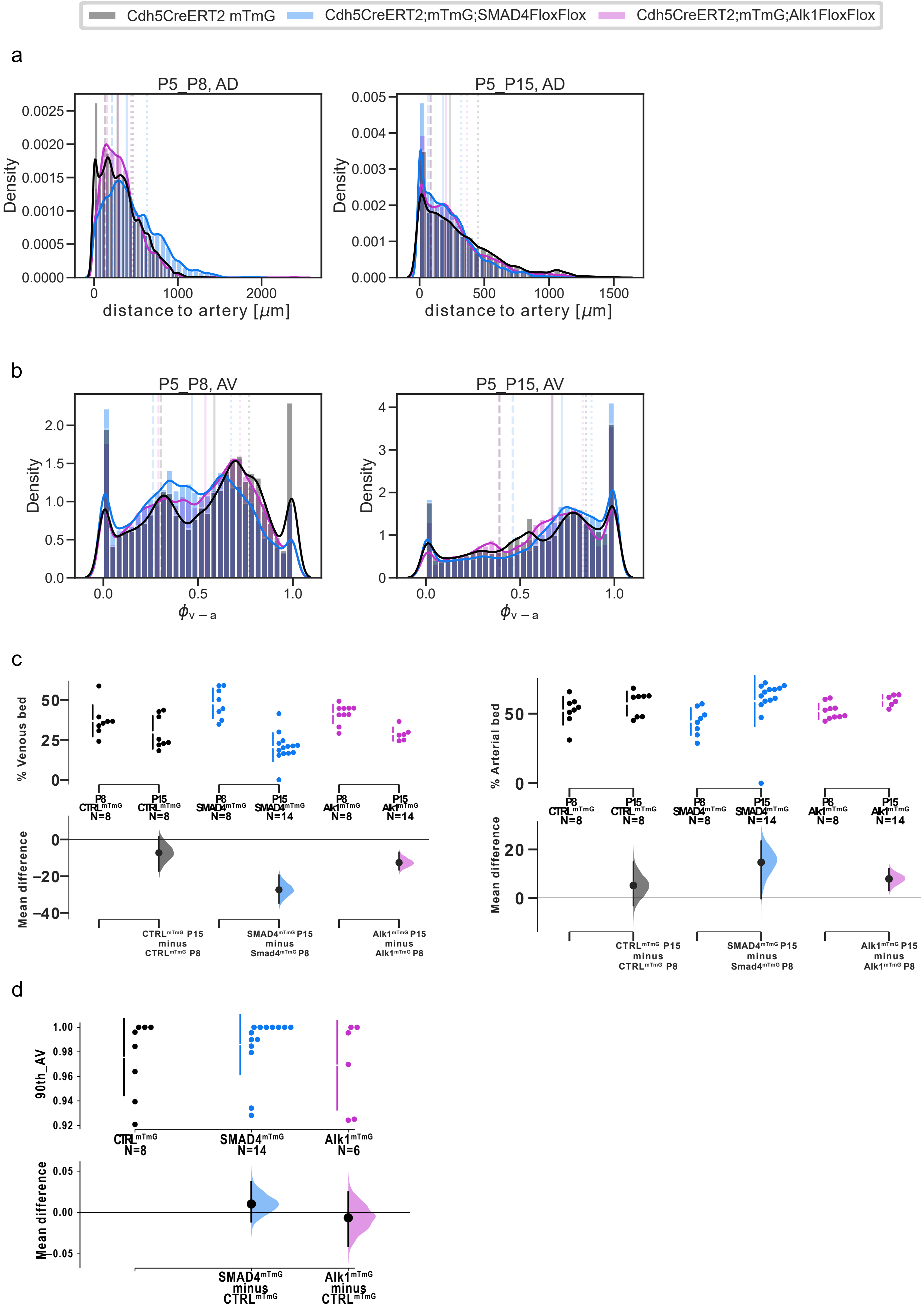

Supplementary figure 4:

Mosaic EC population shift *in vivo*.

a. Density plots of $CTRL^{mTmG}$ (black), $SMAD4^{mTmG}$ (blue) and $Alk1^{mTmG}$ (magenta) in overlay, describing GFP+EC density relative to artery at P8 (left) and P15 (right).

b. Density plots of $CTRL^{mTmG}$ (black), $SMAD4^{mTmG}$ (blue) and $Alk1^{mTmG}$ (magenta) in overlay, describing GFP+EC density in whole mount retinae on the vein (0.0)-artery (1.0) axis.

c. Mean difference statistical analysis of P15 GFP+EC distribution in the venous (left) and arterial (right) bed relative to P8. N=number of retinae per condition.

d. Mean difference statistical analysis of the 90th percentile on the vein-artery axis. N=number of retinae per condition.

Supplementary figure 5

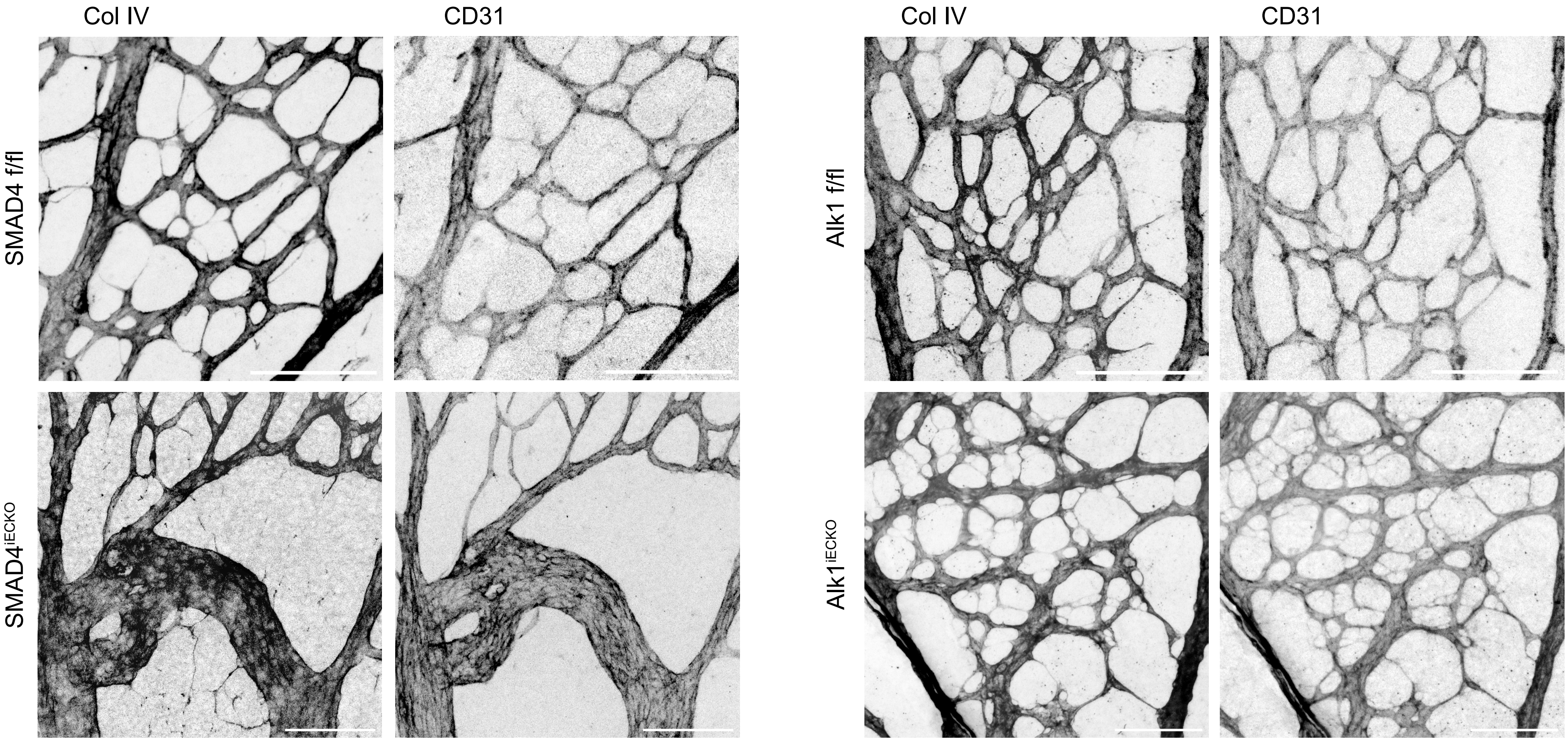

Supplementary figure 5:

Grayscale Col IV and CD31 channel images of enlarged sections of Figure 6. Scale bar 100 µm
